# YOLOv10-Intrusion: An Improved YOLOv10-Based Algorithm for Vehicle Area Intrusion Detection

**DOI:** 10.3390/s26072118

**Published:** 2026-03-29

**Authors:** Chuanyue Jie, Fuyang Ke

**Affiliations:** School of Software, Nanjing University of Information Science and Technology, Nanjing 210044, China; 202312210040@nuist.edu.cn

**Keywords:** object detection, vehicle area intrusion, channel shuffle, one-shot aggregation, dynamic convolution, feature pyramid network, loss function

## Abstract

In intelligent transportation systems and urban traffic management, accurate vehicle area intrusion detection based on surveillance imagery plays a critical role in ensuring road safety and operational efficiency. However, under real-world road surveillance conditions characterized by complex backgrounds, varying illumination, occlusion, and scale variations, mainstream detection algorithms often suffer from high false detection and missed detection rates, limiting their reliability and practical deployment. To address these challenges, this paper proposes YOLOv10-Intrusion, a high-precision vehicle area intrusion detection framework based on an improved version of YOLOv10s. The proposed algorithm incorporates Omni-Dimensional Dynamic Convolution (ODConv) and a custom-designed RCS_M module to enhance feature extraction and fine-grained recognition capability. In addition, a Bidirectional Feature Pyramid Network (BiFPN) is employed to optimize multi-scale feature fusion at the neck level. These improvements collectively reduce false detections and missed detections while improving model recall and mean Average Precision (mAP). Furthermore, the Wise-IoU (WIoU) loss function replaces the original Complete IoU (CIoU) loss to accelerate convergence and stabilize bounding box regression under complex surveillance conditions. A dedicated vehicle area intrusion dataset is constructed from real-world road surveillance footage, covering five vehicle categories across diverse road environments and lighting conditions. Experimental results demonstrate that, compared with the baseline YOLOv10s, YOLOv10-Intrusion achieves improvements of 1.5, 3.3, 3.6, and 2.8 percentage points in Precision, Recall, mAP@0.5, and mAP@0.5:0.95, respectively, and outperforms other mainstream detection algorithms in vehicle area intrusion detection tasks.

## 1. Introduction

With the rapid advancement of deep learning technology, intelligent perception systems have found widespread applications across transportation management, public safety, and infrastructure monitoring [1,2,3]. In urban road management, law enforcement agencies face mounting pressure to replace manual patrols with automated, real-time surveillance solutions capable of detecting traffic violations accurately and efficiently. Vehicle area intrusion—wherein vehicles illegally enter restricted zones such as pedestrian walkways, bicycle lanes, or construction site boundaries—poses significant risks to public safety and presents a persistent challenge in modern road governance [4,5].

Object detection algorithms based on deep learning have demonstrated powerful feature representation and generalization capabilities, making them the dominant paradigm in intelligent traffic surveillance [3]. Among these, the YOLO (You Only Look Once) series of single-stage detectors has achieved widespread adoption due to its favorable balance of speed and accuracy, enabling real-time detection on edge devices [2,6]. Various YOLO-based methods have been proposed for area intrusion detection across different application domains. For instance, Hu et al. [4] proposed MSIA-YOLOv8 for railway obstacle intrusion detection, achieving 97.3% mAP with enhanced robustness in adverse lighting conditions. Zhang et al. [5] integrated YOLOv5-based detection with track area extraction to realize level-based warning for railway obstacles. Dong et al. [7] introduced FPE-YOLO, a YOLOv8-based system achieving 99.14% mAP@0.5 for third-party pipeline intrusion detection under challenging small-object conditions. However, these works primarily address domain-specific intrusion scenarios and rarely tackle the complexity of multi-class vehicle identification under dense traffic, occlusion, and varying illumination conditions.

In the vehicle detection domain, deep learning-based classification and localization methods have been extensively studied. Alnfiai [8] proposed a Lightning Search Algorithm with Deep Transfer Learning for vehicle classification in intelligent transportation systems, demonstrating the importance of accurate multi-class vehicle recognition. Liu et al. [1] developed TIE-LR, a multi-module framework for vehicle identification and tracking from low-resolution CCTV footage using self-supervised learning and distilled YOLO models. Despite significant progress, existing vehicle detection algorithms frequently exhibit high false detection and missed detection rates when applied to practical road surveillance scenarios, particularly when facing visually similar vehicle categories (e.g., trucks and muck trucks), dense overlapping targets, and degraded imaging conditions such as motion blur, night-time low light, or transmission artifacts [6,9].

Multi-domain intrusion detection benchmarks have further revealed the limitations of current approaches. Han et al. [10] introduced the MMID-Bench benchmark, systematically evaluating multi-domain, multi-category intrusion detection algorithms under foggy, rainy, and night-time conditions, highlighting the performance degradation of conventional detectors in adverse environments. Similarly, Huang et al. [11] demonstrated that spatio-temporal feature fusion is critical for accurate intrusion detection in unmanned aerial vehicle (UAV) surveillance scenarios. These findings underscore the need for detection algorithms specifically designed for the practical demands of vehicle area intrusion enforcement, including high precision, strong recall, and robust performance across diverse real-world conditions.

The surveillance scenes monitored by the platform where the proposed algorithm is deployed include multiple corner sections leading to construction sites. In such sections, large vehicles frequently enter the camera view at close range and then gradually move away, resulting in considerable variations in detection scales. Large vehicles appearing at close range to the camera are often incorrectly detected as multiple distinct vehicles. Furthermore, the target road sections covered by the algorithm mainly consist of highways with heavy traffic and field paths with complex backgrounds. In the surveillance view, vehicles in high traffic sections easily occlude each other. At night, strong vehicle headlights cause overexposure, and under rainy, snowy, or hazy weather conditions, images suffer from blurring and color distortion. These issues tend to destroy the integrity of the visual features of vehicle targets, making it difficult for the backbone network of the original YOLO algorithm to extract sufficiently effective and discriminative features.

To address these challenges, this paper proposes YOLOv10-Intrusion, an improved object detection algorithm based on YOLOv10s, tailored for vehicle area intrusion detection in real-world road surveillance environments. The main contributions of this paper are as follows:

- The RCS_M module, a self-designed backbone component, replaces the C2f structure by combining channel-shuffled reparameterized convolution (RCS) with a one-shot aggregation (OSA) strategy and substituting the RepVGG block with MobileOne Block. This design reduces training parameters while enhancing cross-channel information interaction and local feature representation. An additional RCS layer is appended after the second RCS stage to compensate for the accuracy reduction associated with the lighter parameterization, achieving improved precision and mean average precision on the vehicle intrusion dataset.
- Omni-Dimensional Dynamic Convolution (ODConv) is introduced into the neck C2f module (C2f_OD) to replace standard static convolution. By generating four complementary attention weights across spatial, input-channel, output-channel, and kernel dimensions simultaneously, ODConv adaptively highlights discriminative features of visually similar vehicle categories (e.g., trucks versus muck trucks), improving fine-grained recognition capability and suppressing background interference.
- BiFPN (Bidirectional Feature Pyramid Network) is incorporated into the neck to replace the original Path Aggregation Network (PAN)/Feature Pyramid Network (FPN) fusion path. In conjunction, WIoU (Wise-IoU) replaces the Complete IoU (CIoU) loss function in the detection head. BiFPN constructs bidirectional feature flow with learnable fusion weights, adaptively balancing shallow detail and deep semantic features to improve recall in dense and occluded traffic scenes. WIoU dynamically assigns gradient gains based on anchor quality, concentrating regression optimization on normal-quality anchors to accelerate convergence and alleviate the impact of class imbalance.
- A high-quality vehicle area intrusion dataset is constructed from real-world road surveillance footage, covering five vehicle categories across six monitored sections with diverse angles, lighting conditions, and time periods. Field validation across all six monitored sections under both daytime and nighttime conditions confirms the practical effectiveness of the proposed algorithm.

The remainder of this paper is organized as follows. Section 2 reviews related work on vehicle detection, area intrusion detection, and YOLO-based architectural improvements. Section 3 presents the proposed YOLOv10-Intrusion algorithm, covering the technical workflow and all architectural module designs. Section 4 describes the experimental setup and reports results, including ablation studies, comparative experiments, Generalization Experiments, visualization analysis, and field validation. Finally, Section 5 concludes the paper and outlines future research directions.

## 2. Related Work

### 2.1. Vehicle Detection in Traffic Surveillance

Vehicle detection and classification are fundamental tasks in intelligent transportation systems, with broad applications in traffic monitoring, law enforcement, and autonomous driving [3,12]. Early YOLO-based approaches, such as the O-YOLO-v2 model proposed by Han et al. [13] for tiny vehicle detection, demonstrated that multi-scale feature fusion with residual modules significantly improves detection accuracy for small and distant vehicles. Alnfiai [8] proposed a Lightning Search Algorithm with Deep Transfer Learning (LSADTL-VCITS) combining YOLOv5 with Capsule Networks for multi-class vehicle classification, while Liu et al. [1] developed the TIE-LR framework for vehicle identification from low-resolution surveillance footage using self-supervised SimCLR representations, achieving a 17-fold speed improvement over conventional YOLOv8x-based methods. Chaman et al. [14] conducted a comprehensive benchmarking study of YOLOv8 through YOLOv12 in Advanced Driver Assistance Systems (ADAS) scenarios, demonstrating clear generational improvements with YOLOv12 achieving the highest accuracy (mAP@50–95 = 82.2%) across traffic sign recognition, pedestrian detection, and vehicle detection tasks.

A persistent challenge in vehicle detection is maintaining robust performance under degraded image conditions and complex environmental factors. Wang et al. [15] proposed MC-YOLO, integrating MobileNetV2 and CBAM attention for nighttime vehicle detection, achieving 92.75% precision on the BDD100K dataset. Luo et al. [16] introduced IR-YOLO for infrared pedestrian-vehicle detection, employing a dedicated small-target detection head and Focal Generalized IoU (GIoU) loss to achieve 90.1% mAP under low-visibility conditions. Kang et al. [17] proposed YOLO-FA with type-1 fuzzy attention to reduce detection uncertainty in rainy and nighttime scenarios, achieving 8.1% AP50 improvement on the UA-DETRAC dataset. Duan et al. [18] designed DD-YOLO with dual-channel feature extraction and a hybrid pooling pyramid module for blurred vehicle detection, improving mAP@0.5 by 2.7% on KITTI. Du et al. [19] proposed MLE-YOLO based on YOLOv11, combining a Multi-Stage Partial Transformer Module (M-SPTM) with a mixed aggregation network for vehicle and pedestrian detection in adverse weather, achieving 3.0% mAP improvement with 15.8% fewer parameters. Wang et al. [20] proposed Hybrid-YOLO integrating Mamba-based state space modeling and transformer-driven global attention with multi-scale feature fusion, achieving 90.11% mAP@0.5 at 66.3 FPS on the KITTI benchmark.

Dense traffic scenes with frequent occlusion and visually similar vehicles pose additional detection challenges. Liu et al. [21] addressed long-distance, truncation, and occlusion challenges through a Visual Attention Module (VAM) and Feature Reconstruction Module (FRM) inspired by human visual perception, achieving state-of-the-art performance on KITTI. Li et al. [22] proposed YOLO-CCS with coordinate attention and C2f modules for vehicle detection, improving mAP50 by 3.2% over YOLOv5s. Pan et al. [23] proposed LVD-YOLO with EfficientNetv2 backbone and bidirectional feature pyramid for lightweight vehicle detection, reducing FLOPs by 64.6% while maintaining competitive accuracy. Alahdal et al. [24] evaluated multiple YOLO versions in autonomous vehicle environments, highlighting the importance of early detection for diverse road objects. Tang et al. [2] proposed YOLO-Fusion integrating infrared and visible-light images through FusionAttention and Dynamic Fusion modules for multimodal intelligent transportation scenarios. While these works demonstrate the breadth of YOLO-based vehicle detection research, they do not specifically address the multi-class vehicle area intrusion detection scenario under dense, occluded road surveillance conditions with enforcement requirements for high precision and recall.

### 2.2. Area Intrusion Detection

Area intrusion detection aims to identify objects or events that violate spatial boundaries, with applications ranging from railway safety and pipeline monitoring to wildlife protection and road law enforcement. Hu et al. [4] proposed MSIA-YOLOv8, an improved YOLOv8-based detector for railway obstacle intrusion, incorporating multi-scale feature extraction and Frequency Domain Aggregation and Enhancement (FDAE) modules to improve detection robustness under low-light and adverse weather conditions, achieving 97.3% mAP at 137 FPS on a railway dataset. Zhang et al. [5] developed an early warning system integrating YOLOv5-based detection with track area extraction and obstacle severity classification, providing tiered warning outputs for railway intrusion scenarios. Tang et al. [9] introduced YOLO-RCNN, a hybrid framework combining YOLO foreground detection and region of interest (ROI)-aligned RCNN classification for real-time railroad crossing surveillance, achieving 54.15% mAP on a custom railroad crossing dataset and demonstrating deployment feasibility on edge hardware via TensorRT optimization.

For pipeline and infrastructure protection, Dong et al. [7] proposed FPE-YOLO, an enhanced YOLOv8 system for real-time small-object intrusion detection in oil and gas pipeline rights-of-way, employing bio-inspired foveal attention, cross-level fusion, and single-level enhancement modules to achieve 99.14% mAP@0.5 with only a 19.5% increase in computational complexity. Hu et al. [25] proposed a convolutional neural network-based infrared target intrusion detection algorithm combining static target mode analysis (LBP texture) with dynamic multi-frame correlation detection, achieving superior performance in military and intelligent warning applications. Sodhro et al. [6] conducted a systematic real-time evaluation of YOLOv5 and YOLOv8 for human intrusion detection across diverse environmental conditions (luminance variation, indoor/outdoor, simulated weather), revealing that YOLOv8 achieves 99.1% outdoor confidence while adverse weather significantly reduces its effectiveness. Balakrishnan et al. [26] proposed Deep-Track, a field surveillance system combining VGG16-based animal classification with Deep-SORT tracking for real-time wildlife intrusion detection in human-fringe areas, achieving 92.19% accuracy.

Multi-domain benchmarking of intrusion detection algorithms has highlighted performance degradation under challenging environmental conditions. Han et al. [10] introduced MMID-Bench, a comprehensive benchmark for multi-domain multi-category intrusion detection, proposing the MMID-YOLO framework with unsupervised domain adaptation (I-DANN) and diffusion model-based augmentation to improve generalization across normal, foggy, rainy, and night-time city intrusion scenarios. Huang et al. [11] developed a Fused-ConvLSTM-based railway intrusion detection method for UAV surveillance, effectively capturing spatio-temporal features for detecting unknown intruder types in complex aerial scenes with multiple railway regions. Despite this progress, most existing intrusion detection methods focus on specific object categories (persons, animals, or obstacles) and do not address the multi-class vehicle area intrusion scenario under complex road surveillance conditions.

### 2.3. YOLO Architecture Improvements for Detection Tasks

The YOLO series has undergone continuous architectural evolution to improve detection accuracy and efficiency for diverse application scenarios. In backbone and feature extraction design, channel shuffling and reparameterized convolution have emerged as effective strategies for strengthening cross-channel feature interaction. Lu et al. [27] proposed IMV-YOLO incorporating channel shuffle and one-shot aggregation (OSA) strategies for infrared multi-angle vehicle detection under adverse weather, achieving improvements of 4.5% and 7.7% in mAP50 and mAP50-95 over YOLOv11. Li et al. [28] proposed MBS-YOLO with a C2f-Pu module and Multi-Branch Feature Pyramid Network (MB-FPN) for anti-drone detection, reducing parameters by 21.2% while improving mAP@0.5 by 4.5% on Det-Fly. Lu et al. [29] improved YOLOv5 for underground sewer defect detection by incorporating selective kernel attention and a bidirectional cascade feature fusion structure, achieving 4.5% mAP improvement at 69.9 FPS. Fan et al. [30] proposed LUD-YOLO for UAV small object detection, introducing a multi-scale feature fusion mode with upsampling in FPN and dynamic sparse attention mechanisms, demonstrating superior performance on VisDrone2019 and UAVDT datasets. Zhang et al. [31] proposed NOC-YOLO based on YOLOv10n for small-target vehicle detection in aerial infrared images, achieving 79.5% mAP50 on the DroneVehicle dataset by incorporating attention mechanisms and multi-scale feature fusion.

In feature pyramid design, bidirectional and adaptive multi-scale fusion strategies have demonstrated consistent improvements in handling targets of varying scales and densities. Song et al. [32] proposed MEB-YOLO combining BiFPN with ECA attention for complex traffic road vehicle detection on the UA-DETRAC benchmark, demonstrating that bidirectional feature fusion significantly improves multi-scale vehicle detection performance. Liu et al. [33] proposed PV-YOLO replacing PANet with BiFPN in the neck for pedestrian and vehicle detection on BDD100K and KITTI, achieving higher detection accuracy than YOLOv8n with lower computational complexity. Nan et al. [34] proposed MS-YOLO-DLKA combining multi-scale feature extraction (MS-Block) and large convolutional kernels (D-LKA) for LiDAR-camera joint railway obstacle detection, achieving 91% mAP on a custom railway dataset. Chaurasia and Patro [35] introduced YOLO-CSL with a novel Channel and Spatial Attention (CSA) module for rotational object detection in satellite and aerial imagery.

For loss function optimization, improved IoU-based losses have shown consistent benefits in bounding box regression stability, particularly for datasets with class imbalance and dense, occluded targets. Zhou et al. [36] proposed MP-YOLO for dense vehicle detection on the DAIR-V2X dataset, replacing CIoU with WIoU to effectively address the high-overlap characteristics of road targets and improve AP50 by 4.7%. Tahir et al. [37] incorporated WIoU v3 into PV3M-YOLO for UAV-based pedestrian and vehicle detection on VisDrone2019, demonstrating that WIoU’s dynamic gradient allocation reduces the influence of low-quality anchors and improves mAP@0.5 by 3.9% over the baseline. These developments in feature extraction, multi-scale fusion, and loss function design collectively motivate and provide the methodological foundation for the architectural improvements proposed in YOLOv10-Intrusion, which are specifically designed for the demands of vehicle area intrusion detection in real-world road surveillance environments.

## 3. Materials and Methods

### 3.1. Technical Workflow

**Figure 1:**
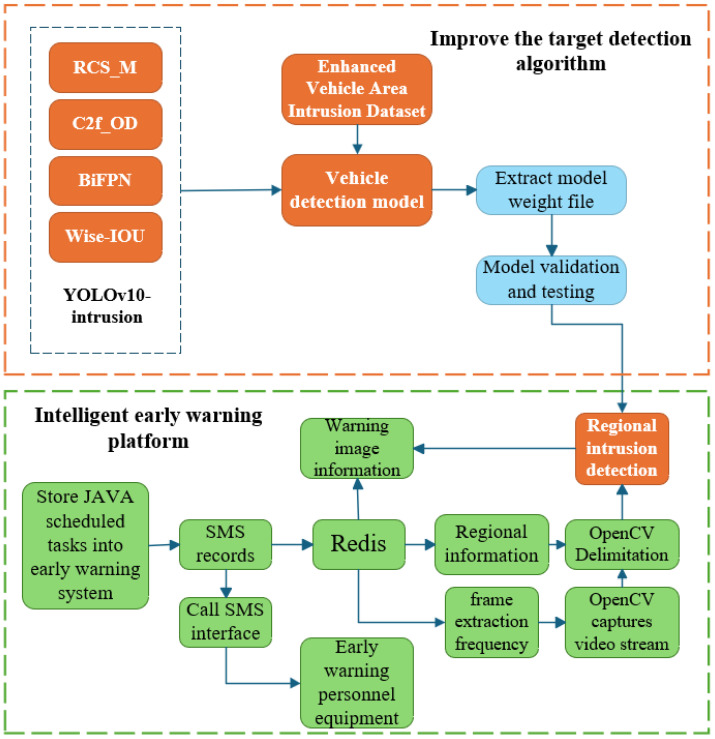
Technical workflow of the vehicle area intrusion detection system.

The overall technical workflow of the proposed system is illustrated in Figure 1. The system consists of two main components: the vehicle area intrusion dataset construction and the YOLOv10-Intrusion detection algorithm. Live video streams are acquired from surveillance cameras via the RTSP (Real-Time Streaming Protocol), and OpenCV is used to extract frames at a configured sampling rate and to delineate detection zones within each frame. The YOLOv10-Intrusion algorithm is then applied to classify and localize vehicles within the designated area. The detection model underwent multiple rounds of field testing before operational deployment.

### 3.2. YOLOv10 Architecture Overview

The YOLO series represents the dominant paradigm in single-stage object detection, offering superior detection accuracy and inference speed for real-time applications. To eliminate the inference latency introduced by Non-Maximum Suppression (NMS) post-processing, YOLOv10 employs a consistent dual-assignment training strategy that achieves NMS-free inference while simultaneously optimizing model efficiency and detection accuracy. YOLOv10 further incorporates a partial self-attention module that substantially enhances feature extraction and multi-scale representation capability, making it particularly suitable as a lightweight baseline for resource-constrained real-time detection tasks.

YOLOv11 introduced the C3k2 module to replace the C2f module in the backbone and neck, decomposing the original large convolution into two smaller convolutions to reduce processing time. A Cross-Stage Partial Spatial Attention (C2PSA) module was appended after the Spatial Pyramid Pooling Fast (SPPF) module to enhance spatial attention and improve detection accuracy in regions of interest. YOLOv12 further extends this by introducing area attention, which partitions feature maps into multiple non-overlapping regions to maintain large receptive fields while avoiding the computational overhead of complex window partitioning operations, effectively integrating attention mechanisms into the YOLO framework. However, the relatively constrained parameter capacity of YOLOv12 limits its representational power for complex multi-scale feature patterns.

Considering the lightweight design philosophy, excellent detection performance, and NMS-free inference pipeline of YOLOv10s, it is selected as the baseline model for vehicle area intrusion detection in this paper. Recent works have further validated YOLOv10 as an effective baseline for vehicle detection; for instance, Zhang et al. [31] demonstrated that YOLOv10n achieves competitive performance for aerial infrared small-target vehicle detection, confirming the model’s adaptability to challenging real-world scenarios.

### 3.3. YOLOv10-Intrusion Architecture

**Figure 2:**
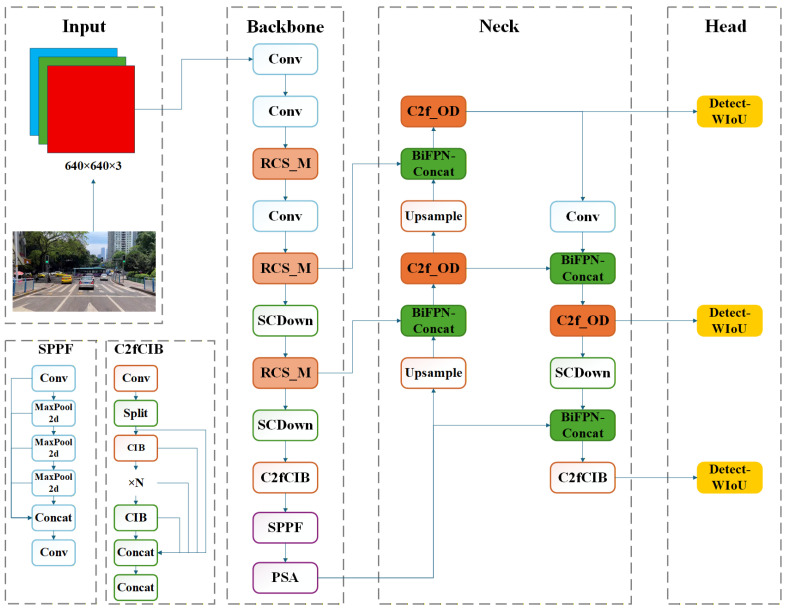
Architecture of YOLOv10-Intrusion. Modified modules are highlighted in dark color.

The proposed YOLOv10-Intrusion algorithm adopts YOLOv10s as the baseline. As illustrated in Figure 2, the backbone C2f modules are replaced with the proposed RCS_M module, while standard convolution in the neck C2f module is replaced with ODConv to form the C2f_OD module. BiFPN is introduced in the neck to optimize multi-scale feature fusion, and the CIoU loss function in the detection head is replaced with WIoU. Modified modules are highlighted in dark color in the figure.

The RCS_M module, C2f_OD module, BiFPN feature fusion structure, and WIoU loss function used in YOLOv10s form a complete collaborative mechanism that covers feature extraction, feature enhancement, multi-scale feature fusion, and bounding box regression, jointly solving the challenging problems of large target scale changes, insufficient feature extraction capabilities, and class imbalance in vehicle area intrusion detection. The RCS_M module in the backbone enhances local features and facilitates cross-channel information interaction through structural reparameterization, channel shuffle, and one-shot aggregation (OSA), while introducing only a small increase in parameters and computational cost. Compared with the original C2f module, RCS_M provides more complete and discriminative features for subsequent modules, thereby solving the difficulty of feature extraction in complex environments. In the neck network, the C2f_OD module integrated with ODConv adaptively highlights critical vehicle structures, including contours and textures, via multi-dimensional dynamic attention; suppresses background interference; and makes the basic features output by RCS_M more distinguishable. BiFPN enables bidirectional delivery and weighted fusion of deep semantic information and shallow detailed information, allowing the fine-grained features extracted by the preceding two modules to be fully utilized across all scales. This makes the model more adaptable to large variations in target scale, ranging from close-range large trucks to distant small cars, and reduces false recognition. Finally, the WIoU loss dynamically allocates gradients according to anchor quality, reduces interference from low-quality anchors, accelerates model convergence, and mitigates class imbalance. In this way, the high-quality features refined by the backbone and neck can be more stably converted into accurate detection results. When deployed on the intelligent enforcement platform, the improved model effectively detects overlapping vehicles and significantly reduces false recognition of close-range large vehicles compared with the original YOLOv10s. Thus, it is highly suitable for the target road sections monitored by the platform.

#### 3.3.1. RCS_M Module

In road surveillance scenarios with high traffic density, mutual occlusion between vehicles frequently results in incomplete or partially visible feature regions. Furthermore, large vehicles such as trucks and muck trucks share highly similar appearance characteristics, leading to frequent inter-class misclassification. To address these problems, the backbone structure is improved by introducing the self-designed RCS_M module to replace the C2f structure, strengthening cross-channel information exchange and local feature representation capability.

**Figure 3:**
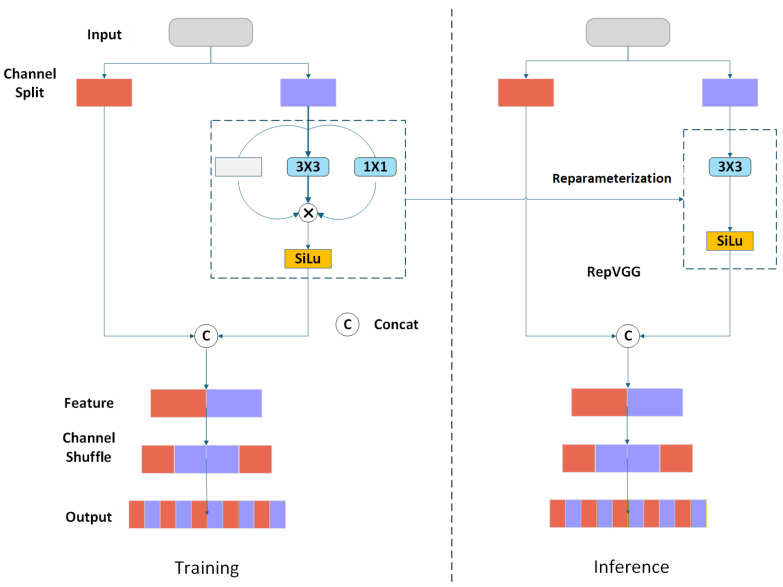
Structure of the RCS module.

The RCS (Reparameterized Channel Shuffle) module maintains a multi-branch structure during training, consisting of a 1×1 dimensionality-reduction convolution, a 3×3 convolution, and an identity connection. Channel shuffling has been shown to be an effective strategy for strengthening cross-channel feature interaction in vehicle detection backbones [27]; combined with the multi-branch structure, it significantly enhances cross-channel information flow during training. The training-time output of the RCS module is expressed as:

(1) $$y_{RCS}=W_{1\times 1}*x+W_{3\times 3}*x+x$$

where $W_{1\times 1}$ and $W_{3\times 3}$ denote the 1×1 and 3×3 convolutional kernels, respectively, ∗ denotes the convolution operation, and x is the input feature map. During inference, structural reparameterization merges all branches into a single equivalent 3×3 convolution:

(2) $$y_{RCS}=W_{eq}*x,\;W_{eq}=W_{1\times 1}^{\prime }+W_{3\times 3}+W_{id}$$

where $W_{1\times 1}^{\prime }$ is the zero-padded 3×3 equivalent of the 1×1 kernel and $W_{id}$ is the identity mapping expressed as a 3×3 kernel, reducing computational overhead while preserving multi-branch feature learning capability and improving feature representation in dense and occluded regions. The structure of the RCS module is shown in Figure 3.

**Figure 4:**
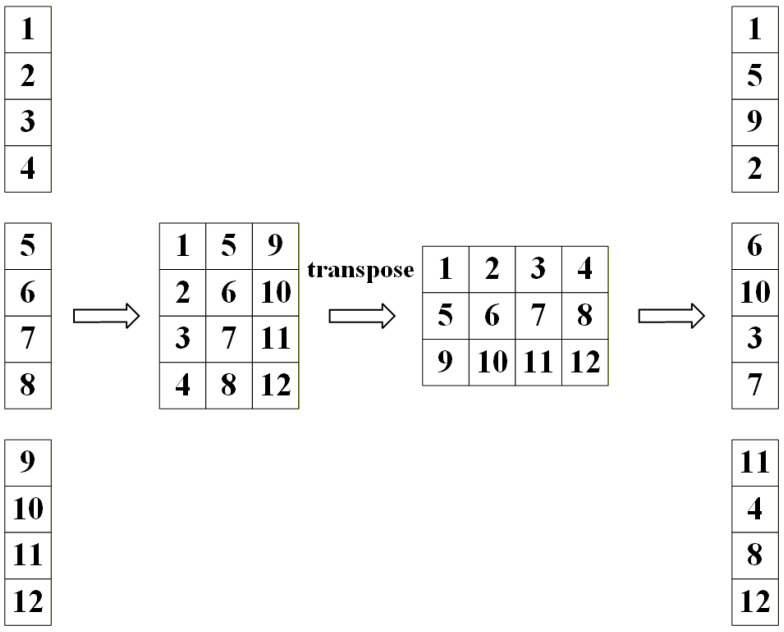
Channel Shuffle.

The principle of Channel Shuffle is to unfold the input feature map into a matrix, then transpose the resulting matrix, and finally flatten the transposed channels to complete the final channel shuffle. The specific operation is shown in Figure 4. Each piece of data is shuffled through channels and then aggregated using Concat, followed by a RepVGG operation.

OSA (One-Shot Aggregation) is a complementary module that aggregates all intermediate features through a single global operation at the module output, maintaining cross-channel information exchange while improving network efficiency relative to dense connectivity strategies. Input features from the preceding layer are first dimensionality-reduced by the RepVGG block and then routed through two parallel paths: a direct skip connection and a sequential processing path through two stacked RCS modules that extract hierarchical features at different depths. The outputs of both paths are merged via channel shuffling, producing representations with stronger cross-scale interaction capability.

Although the original RCS-OSA module yields remarkable accuracy gains for the model, it suffers from excessive parameters. In addition, the features produced by two RCS layers still result in misidentification in practical deployment. Inspired by the lightweight MobileOne network, we revise the RCS-OSA module by replacing its RepVGG block with the MobileOne Block. In the RCS_M module, input features first go through channel dimension reduction via the MobileOne Block and are then split into two branches: a shortcut branch and an RCS processing branch. The RCS branch adopts three consecutive RCS operations to mine deeper-level features, laying a richer feature foundation for the subsequent channel shuffle. The features processed by the RCS modules are then fused with the shortcut features from the MobileOne Block via channel shuffle, which breaks information barriers between channels, enables free feature flow across different groups, and allows the model to capture more comprehensive representations. Finally, the OSA (One-Shot Aggregation) strategy is adopted to globally aggregate the shuffled features in one step, outputting enhanced multi-scale vehicle features. Compared with the original RCS-OSA module, RCS_M utilizes the MobileOne Block instead of the RepVGG block for channel downsampling. Its depthwise separable convolutions reduce both parameters and computational cost. Meanwhile, an additional third RCS layer is appended to further mine deep features and compensate for the accuracy loss caused by the MobileOne Block. This operation introduces no significant extra computation or parameter overhead, thus preserving the overall lightweight advantage of the model.

**Figure 5:**
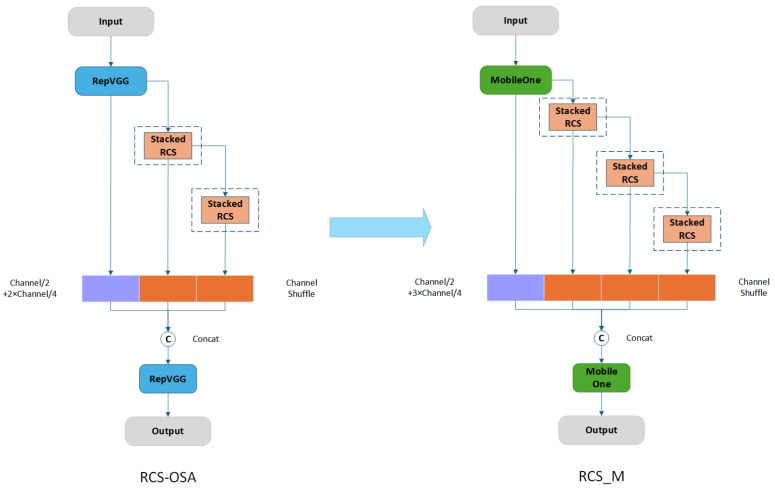
Comparison of RCS-OSA and the proposed RCS_M module.

When integrated into the backbone network, the improved RCS_M module reduces the parameters by 1.7 M and improves mAP@0.5 by 0.8 percentage points relative to the original RCS-OSA module. In practical deployment, the RCS_M module correctly identifies vehicles that were misclassified by the original algorithm, leading to a significant reduction in large-vehicle misidentification in surveillance scenarios. The improvement from RCS-OSA to RCS_M is illustrated in Figure 5.

#### 3.3.2. ODConv Module (C2f_OD)

Road surveillance environments involve multiple vehicle categories with complex and fine-grained appearance features. The fixed weights of standard convolutions are incapable of dynamically adjusting their responses to varying feature distributions, limiting the model’s ability to distinguish visually similar vehicle categories. To address this limitation, Omni-Dimensional Dynamic Convolution (ODConv) is introduced to replace standard convolution in the neck C2f module, optimizing the feature extraction process.

**Figure 6:**
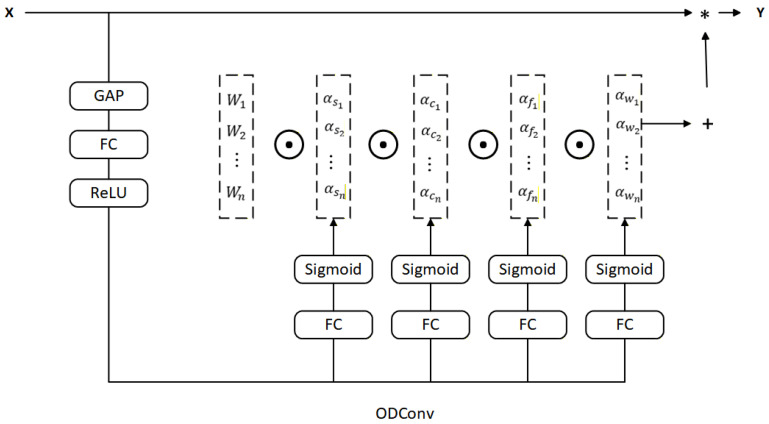
Overview of the ODConv module with four complementary attention dimensions.

ODConv generates four complementary attention weights across spatial, input-channel, output-channel, and kernel dimensions simultaneously. This multi-dimensional dynamic adjustment enables the model to highlight discriminative features such as vehicle contour, texture channels, and spatial regions while suppressing irrelevant background features. An overview of the ODConv module is illustrated in Figure 6. The ODConv formulation is given in Equations (3) and (4):

(3) $$y=\left(\sum_{i=1}^{n}\beta_{i}\right)*x$$

(4) $$\beta_{i}=\alpha_{si}⊙\alpha_{ci}⊙\alpha_{fi}⊙\alpha_{wi}⊙W_{i}$$

where *x* denotes the input feature; *y* denotes the output feature; $\beta_{i}$ represents the dynamic weight of the *i*-th convolutional kernel; ⊙ denotes element-wise multiplication along different dimensions of the kernel space; $W_{i}$ is the *i*-th convolutional kernel; and $\alpha_{si}$, $\alpha_{ci}$, $\alpha_{fi}$, and $\alpha_{wi}$ represent attention weights along the spatial, input-channel, output-channel, and kernel dimensions, respectively.

In the attention generation process, ODConv first applies Global Average Pooling (GAP) to each input channel to produce a compact channel-wise descriptor $g\in R^{C}$:

(5) $$g_{c}=\frac{1}{H\times W}\sum_{h=1}^{H}\sum_{w=1}^{W}x_{c}\left(h,\;w\right)$$

where *H* and *W* are the spatial dimensions of the input feature and $x_{c}\left(h,w\right)$ is the feature value at position (h,w) in channel *c*. A fully connected (FC) layer subsequently reduces dimensionality and performs global feature combination, followed by ReLU activation. The spatial attention weight $\alpha_{si}$ is then generated via Sigmoid activation, while the input-channel, output-channel, and kernel attention weights are generated via Softmax:

(6) $$\alpha_{si}=\sigma \;\left(f_{s}\left(g\right)\right)$$

(7) $$\alpha_{ci}=\mathrm{softmax}\;\left(f_{c}\left(g\right)\right),\;\alpha_{fi}=\mathrm{softmax}\;\left(f_{f}\left(g\right)\right),\;\alpha_{wi}=\mathrm{softmax}\;\left(f_{w}\left(g\right)\right)$$

where $f_{s}$, $f_{c}$, $f_{f}$, $f_{w}$ are the corresponding FC projection layers and σ(·) denotes the Sigmoid function. The convolutional kernels $W_{i}$ are then combined with their respective attention weights through element-wise multiplication, and the weighted results are summed to produce the dynamically adjusted output feature *y*.

**Figure 7:**
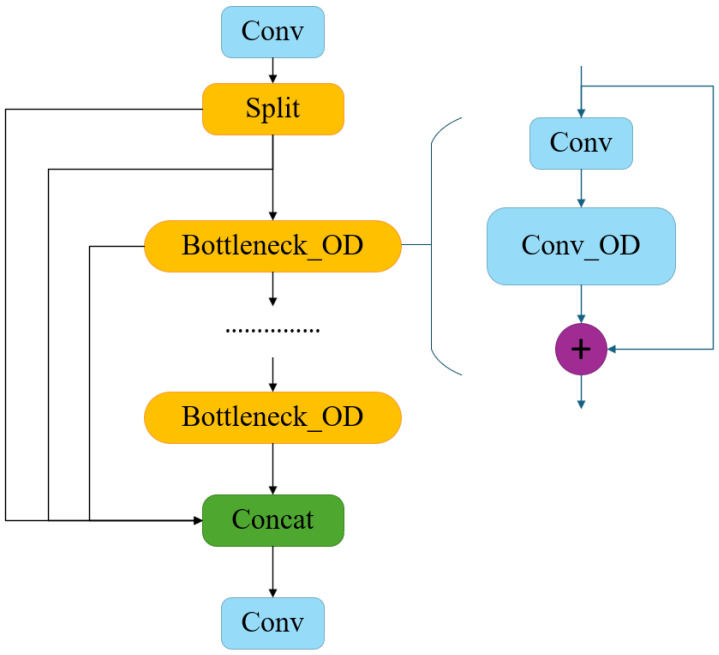
Structure of the C2f_OD module with ODConv replacing standard convolution.

The four complementary attention weights $\alpha_{si}$, $\alpha_{ci}$, $\alpha_{fi}$, and $\alpha_{wi}$ enable ODConv to differentially emphasize vehicle-discriminative features: highlighting vehicle contour and texture channels for compact cars, adapting kernels to distinctive shape characteristics of large vehicles (muck trucks versus trucks), focusing attention on vehicle-surrounding regions, and suppressing road background interference, thereby improving detection accuracy for partially occluded vehicles. By replacing standard convolution in the C2f module with ODConv, the improved C2f_OD module (shown in Figure 7) enables the network to adaptively focus on key vehicle regions during feature extraction.

#### 3.3.3. BiFPN Feature Pyramid

Traditional YOLO models employ PAN/FPN structures that perform feature transfer through simple upsampling, downsampling, and element-wise addition, without adaptive adjustment of feature scale contributions. In road surveillance environments with dense and mutually occluded vehicles, this design is prone to losing critical information across scales. To resolve this issue, the Bidirectional Feature Pyramid Network (BiFPN) [32,33] is introduced to optimize the neck network’s feature fusion process.

BiFPN extends conventional multi-scale feature fusion by breaking unidirectional feature flow constraints and constructing bidirectional feature fusion paths. Through skip connections, high-level semantic information is propagated downward to enrich shallow feature maps with contextual awareness, while low-level spatial detail features are propagated upward to reinforce the localization capability of higher-level representations. BiFPN further introduces learnable fusion weights to adaptively calibrate the contribution of each feature scale. The weighted top-down fusion at intermediate node $P_{l}^{td}$ is formulated as:

(8) $$P_{l}^{td}=\frac{w_{1}\cdot P_{l}+w_{2}\cdot \mathrm{Resize}\left(P_{l+1}\right)}{w_{1}+w_{2}+\epsilon }$$

**Figure 8:**
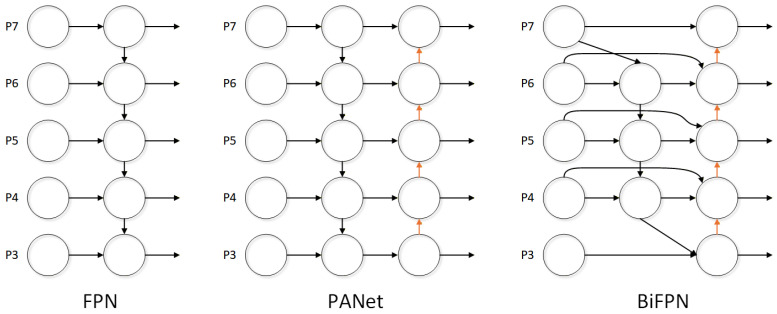
Structural comparison of FPN, PAN, and BiFPN.

The output node $P_{l}^{out}$ is subsequently obtained by fusing the original node, the top-down intermediate, and the upsampled lower-level output:

(9) $$P_{l}^{out}=\frac{w_{1}^{\prime }\cdot P_{l}+w_{2}^{\prime }\cdot P_{l}^{td}+w_{3}^{\prime }\cdot \mathrm{Resize}\left(P_{l-1}^{out}\right)}{w_{1}^{\prime }+w_{2}^{\prime }+w_{3}^{\prime }+\epsilon }$$

where $w_{i}$ and $w_{i}^{\prime }$ are learnable non-negative fusion weights, $P_{l}$ denotes the feature map at pyramid level *l*, and $\epsilon =10^{-4}$ is a small constant for numerical stability. Compared with FPN and PAN structures, the BiFPN-augmented neck significantly improves model recall and mean average precision and reduces the missed detection rate for partially obscured targets in real-world road environments. A structural comparison of FPN, PAN, and BiFPN is shown in Figure 8.

#### 3.3.4. WIoU Loss Function

IoU (Intersection over Union) is a standard metric for evaluating the localization accuracy of object detection predictions. The basic IoU loss function $L_{\mathrm{IoU}}$ is defined as:

(10) $$\mathrm{IoU}=\frac{P\cap G}{P\cup G}$$

(11) $$L_{\mathrm{IoU}}=1-\mathrm{IoU}$$

where *P* denotes the predicted bounding box and *G* denotes the ground-truth bounding box. The IoU loss is zero when boxes perfectly overlap and one when they are completely disjoint. However, the original IoU loss suffers from two key limitations: it provides no gradient information when predicted and ground-truth boxes do not overlap, and identical IoU values may correspond to different relative box configurations, limiting its discriminative power for precise localization.

To overcome these shortcomings, CIoU extends IoU by incorporating center-point distance and aspect ratio consistency penalties:

(12) $$L_{\mathrm{CIoU}}=1-\mathrm{IoU}+\frac{\rho^{2}\left(b,\;b^{g}\right)}{c^{2}}+\alpha v$$

where $\rho^{2}\left(b,b^{g}\right)$ is the squared Euclidean distance between the centers of the predicted box *b* and ground-truth box $b^{g}$, *c* is the diagonal length of the minimum enclosing box, and the aspect ratio consistency term *v* and its trade-off coefficient α are:

(13) $$v=\frac{4}{\pi^{2}}\;{\left(\arctan\frac{w^{g}}{h^{g}}-\arctan\frac{w}{h}\right)}^{\;2},\;\alpha =\frac{v}{(1-\mathrm{IoU})+v}$$

where *w*, *h* and $w^{g}$, $h^{g}$ are the width and height of the predicted and ground-truth boxes, respectively. Although CIoU provides richer geometric supervision than plain IoU, during field testing on the monitored road sections, the original CIoU loss was found to produce suboptimal gradient weight assignments, leading to bounding box drift and redundant box generation that degraded localization precision. The Wise-IoU (WIoU) loss function [36,37] dynamically adjusts loss weights and gradient allocation, assigning higher weights to overlapping or occluded vehicle samples to strengthen bounding box regression optimization for these challenging cases. Its gradient gain allocation strategy also alleviates gradient explosion and reduces the generation of invalid gradients without incurring additional computational cost. The WIoUv1 formulation is given in Equations (14) and (15):

(14) $$L_{\mathrm{WIoUv}1}=R_{\mathrm{WIoU}}\cdot L_{\mathrm{IoU}}$$

(15) $$R_{\mathrm{WIoU}}=\exp\left(\frac{{\left(x-x_{g}\right)}^{2}+{\left(y-y_{g}\right)}^{2}}{{\left(W_{s}^{2}+H_{s}^{2}\right)}^{*}}\right)$$

where $W_{s}$ and $H_{s}$ are the width and height of the minimum enclosing box, the superscript ∗ denotes detachment from the computation graph to prevent $R_{\mathrm{WIoU}}$ from generating gradients that impede convergence, (x,y) is the center coordinate of the predicted box, and $\left(x_{g},y_{g}\right)$ is the center coordinate of the ground-truth box. WIoUv1 constructs distance attention through $R_{\mathrm{WIoU}}$ and $L_{\mathrm{IoU}}$, enhancing the importance of average-quality predicted boxes while weakening geometric penalties when predicted and ground-truth boxes are highly overlapping.

WIoU employs an outlier degree variable β to measure anchor box quality. Smaller outlier degree indicates higher anchor quality. To concentrate bounding box regression on normal-quality anchors, WIoU adopts a gradient gain assignment strategy that assigns smaller gains to both low and high outlier degree anchors, reducing the negative influence of low-quality samples on model training. The final WIoU loss function is:

(16) $$L_{\mathrm{WIoU}}=\frac{\beta }{\delta \cdot \alpha^{\beta -\delta }}\cdot R_{\mathrm{WIoU}}\cdot L_{\mathrm{IoU}}$$

where δ is a hyperparameter used to dynamically regulate the bounding box regression optimization direction and gradient distribution in the detection task. In the initial phase of dataset construction, the vehicle area intrusion dataset suffered from significant class imbalance, with muck trucks and vans substantially underrepresented compared to other categories. WIoU’s robustness to low-quality samples, combined with its dynamic gradient gain allocation mechanism, partially alleviates this class imbalance problem.

## 4. Experiments

### 4.1. Dataset

The construction of the vehicle area intrusion dataset proceeded through three stages of iterative collection and refinement. In the initial stage, 3000 passenger car images sourced from public datasets and 500 muck truck images captured at adjacent construction sites constituted the preliminary dataset, primarily used to evaluate the algorithm’s baseline detection capability for small and large vehicle categories. In the subsequent collection phase, an additional 2000 images covering van, truck, and tricycle categories were gathered from roads and pedestrian areas. To address class imbalance caused by the disproportionate number of car images, approximately 60% of low-quality (duplicate, blurred) car images were filtered, and the WIoU loss function was introduced concurrently. Data augmentation including random cropping was applied to underrepresented van and tricycle samples. In the final refinement stage, category-specific supplementation was performed for truck and muck truck data, including close-range and long-range examples to enrich vehicle detail and small-target representation. After the online platform began operation, background images without any target vehicles and images with transmission artifacts (i.e., frame corruption caused by signal interference) were added as negative samples to suppress false alerts.

**Figure 9:**
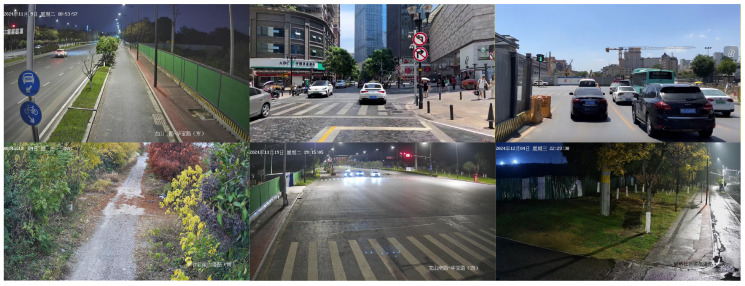
Sample images from the vehicle area intrusion dataset. (The text in the picture represents the time and location of the shooting).

The complete dataset encompasses five vehicle categories: Car, Van, Muck Car, Truck, and Tricycle, covering multiple road environments including urban roads, pedestrian walkways, rural paths, and construction site entrances. A total of 6530 images were collected, of which 80% were captured by on-site photography and surveillance cameras, and 20% were selected from online public datasets. The dataset encompasses vehicles captured across diverse viewing angles and under varying illumination conditions across the full 24-h daily cycle. Data augmentation including Mosaic, random cropping, contrast adjustment, and geometric transformation was applied to selected subsets. Annotation was performed using Roboflow, yielding a total of 12,460 labeled vehicle instances. The dataset is partitioned into 5674 training images, 756 validation images, and 100 test images supplemented by several video segments for qualitative evaluation. As the proposed algorithm is specifically designed for real-time vehicle area intrusion detection in continuous surveillance video streams, the 100 images in the test set are only used for preliminary quantitative evaluation. The primary assessment of model detection performance is conducted on real surveillance video streams collected from the target road sections under monitoring. Field validation is performed using over 24 h of real-world monitoring footage, which is highly consistent with the actual deployment scenario of the model. Category-level instance counts are provided in Table 1. Representative sample images from the dataset are shown in Figure 9.

Compared with generic vehicle datasets, the proposed dataset possesses several distinctive characteristics. The collection encompasses near-full-angle vehicle images together with a diverse set of hard examples including mutually occluded vehicles and motion-blurred high-speed vehicles. The dataset spans six monitored road sections ranging from rural grass-bordered paths to urban roads, with 24-h temporal coverage that provides complex background variation for improving cross-scenario generalization. Moreover, the dataset undergoes continuous iterative refinement driven by real-world deployment observations, incorporating newly discovered failure modes such as rain and snow conditions and transmission artifact frames, which provides robust data support for ongoing algorithm improvement.

### 4.2. Experimental Setup

#### 4.2.1. Implementation Details

All training and testing experiments were conducted on the same workstation running Windows 11, with all models trained for 300 epochs using a batch size of 16, an initial learning rate of 0.01 with cosine annealing decay, and an input image resolution of 640×640. The software and hardware configuration is listed in Table 2.

The input data were resized to 640 × 640 pixels, and the Stochastic Gradient Descent (SGD) algorithm was employed as the optimizer. The performance of the network model ceased to improve after 200 training epochs; thus, the number of training epochs was set to 200 in this paper. The experimental parameters is listed in Table 3.

#### 4.2.2. Comparison Methods

To comprehensively evaluate the proposed method, YOLOv10-Intrusion is compared against the following representative baseline detectors. Faster R-CNN [38] is a classical two-stage detector that uses a Region Proposal Network (RPN) to generate candidate regions followed by an ROI-aligned classification branch. SSD (Single Shot MultiBox Detector) [39] is a one-stage multi-scale anchor-based detector that directly regresses class scores and box offsets from multiple feature map levels. YOLOv8s [40] is the small-scale variant of YOLOv8, adopting a C2f backbone and a decoupled detection head with anchor-free regression. YOLOv11s [41] introduces C3k2 and C2PSA modules for enhanced multi-scale spatial attention. YOLOv12s [42] incorporates an area attention mechanism that partitions feature maps into non-overlapping regions for improved large receptive field modeling. RT-DETR-L is an advanced transformer detector that achieves fast, accurate real-time object detection through dynamic sparse attention and efficient end-to-end decoding. Deformable DETR extends the DETR framework by introducing deformable attention mechanisms, which adaptively sample informative regions from feature maps to alleviate the high computational cost of standard self-attention [43,44]. This design enables Deformable DETR to effectively handle dense object detection and complex scenes, providing a strong transformer-based baseline for comparison. YOLO-LCR [45] is a YOLO-based detector incorporating the Reparameterized Channel Shuffle (RSC) mechanism, serving as a direct architectural reference for the proposed RCS_M module. TSA-YOLO [46] is a YOLO variant specifically designed for vehicle detection under variable illumination conditions, providing a domain-targeted baseline for nighttime and adverse-lighting scenarios. All comparison models were trained and evaluated under identical experimental configurations on the vehicle area intrusion dataset.

#### 4.2.3. Evaluation Metrics

The main evaluation metrics adopted in this paper are precision (P), recall (R), mAP@0.5, and mAP@0.5:0.95, defined as follows:

Precision (P) measures the proportion of correctly predicted positive samples among all samples predicted as positive:

(17) $$P=\frac{TP}{TP+FP}$$

Precision directly quantifies the proportion of valid enforcement alerts among all triggered alerts, reflecting whether intrusion warnings issued by the platform correspond to actual violations. High precision is essential for the practical usability of the detection system.

Recall (R) measures the proportion of correctly detected positive samples among all actual positive samples:

(18) $$R=\frac{TP}{TP+FN}$$

where TP denotes true positives, FP denotes false positives, and FN denotes false negatives. In vehicle area intrusion detection, recall quantifies the algorithm’s capacity to detect all genuine intrusion events without omission. High recall is a prerequisite for reliable enforcement monitoring, particularly under challenging conditions such as target occlusion, nighttime low illumination, and adverse weather.

Mean average precision (mAP) reflects the average detection accuracy across all categories. The AP for each category is computed as the area under the precision-recall (PR) curve:

(19) AP=∫PdR

(20) $$\mathrm{mAP}=\frac{1}{K}\sum_{i=1}^{K}{\mathrm{AP}}_{i}$$

where *K* is the total number of categories (5 in this dataset). mAP@0.5 uses a fixed IoU threshold of 0.5. mAP@0.5:0.95 provides a more stringent localization evaluation by averaging mAP across ten uniformly spaced IoU thresholds:

(21) $$\mathrm{mAP}@0.5:0.95=\frac{1}{10}\sum_{t\;\in \;\{0.50,\;0.55,\;\ldots ,\;0.95\}}\mathrm{mAP}\left(t\right)$$

The $F_{1}$ score combines precision and recall into a single balanced indicator of detection performance:

(22) $$F_{1}=\frac{2\cdot P\cdot R}{P+R}$$

A high $F_{1}$ score reflects a favorable trade-off between false alarms and missed detections, which is particularly relevant for enforcement applications that demand both precision and recall.

### 4.3. Results and Analysis

#### 4.3.1. Comparison Experiments

To validate the superiority of YOLOv10-Intrusion for vehicle area intrusion detection, comparison experiments were conducted against the seven baseline detectors described in Section 4.2.2 under identical experimental settings. Results are shown in Table 4.

YOLOv10-Intrusion achieves mAP@0.5 values that are 15.4, 13.3, 4.5, 3.8, 4.2, 5.1, and 8.8 percentage points higher than Faster R-CNN, SSD, YOLOv8s, YOLOv11s, YOLOv12s, RTDETR-L and Deformable DETR respectively, with mAP@0.5:0.95, precision, and recall all outperforming compared methods. The algorithm also outperforms TSA-YOLO and YOLO-LCR, confirming that the proposed combination of RCS_M, ODConv, BiFPN, and WIoU yields consistent and complementary improvements for the vehicle area intrusion detection task, meeting the demands of real-time, high-precision enforcement monitoring under real-world road surveillance conditions.

#### 4.3.2. Generalization Experiment

To further verify the effectiveness of the proposed algorithm, we conducted generalization experiments on three public datasets: KITTI, VOC2007, and COCO. We selected 3000 images from KITTI, 2500 images from VOC2007, and 2800 images from COCO for the generalization experiments. Since the target categories in this study are car, van, truck, muck car, and tricycle, we only retained images containing the above five vehicle categories from the public datasets and removed the corresponding annotations of irrelevant classes. The experimental environment and parameter settings were kept consistent with those described above. Results are shown in Table 5.

For model detection performance, on the KITTI dataset, YOLOv10-Intrusion improves the mAP@0.5 by 2.4 percentage points and mAP@0.5:0.95 by 1.8 percentage points compared with YOLOv10s. On the VOC2007 dataset, YOLOv10-Intrusion increases mAP@0.5 by 1.7 percentage points and mAP@0.5:0.95 by 2.6 percentage points. On the COCO dataset, YOLOv10-Intrusion boosts mAP@0.5 by 5.4 percentage points and mAP@0.5:0.95 by 3.8 percentage points. The experimental results demonstrate that the proposed algorithm achieves superior detection performance on various public datasets and exhibits strong generalization ability, effectively reducing false detection and missed detection rates.

#### 4.3.3. Ablation Study

YOLOv10-Intrusion incorporates four architectural improvements over the baseline YOLOv10s: the RCS_M module in the backbone, the C2f_OD module replacing the original C2f in the neck, BiFPN for neck-level feature fusion, and WIoU replacing CIoU in the detection head. To evaluate the individual contribution of each modification and assess the effectiveness of the overall design, ablation experiments were conducted on the custom dataset under consistent hardware, software, and training configurations across all experiment groups. Results are shown in Table 6.

Experiment 1 shows that the C2f_OD module adaptively weights visual features and focuses on the detailed and discriminative regions of vehicles, enabling the model to recognize hard samples that are difficult to detect using the original model. This brings a 1.3% improvement in recall and enhances the fine-grained recognition capability in practical applications, which better meets the expected requirements of law enforcement departments. Experiment 2 demonstrates that the RCS_M module improves the model performance comprehensively. Compared with the original C2f module, the RCS_M module extracts more complete feature information and enhances cross-channel information interaction in the backbone network, providing more discriminative features for subsequent modules. Consequently, the overall performance of the model is improved, especially in terms of mAP@0.5, which is increased by 2.3 percentage points. Experiment 3 indicates that the BiFPN module complements shallow detailed information and deep semantic information through bidirectional weighted feature fusion, which improves the detection accuracy of the model under scenarios of drastic scale variation and dense occlusion. Specifically, the recall and mAP@0.5 are increased by 1.6 and 0.9 percentage points, respectively. Experiment 4 confirms that WIoU loss function reduces the interference caused by background clutter and low-quality anchors by dynamically adjusting gradient gains, thus improving the bounding box regression accuracy. This leads to a 2.5% improvement in recall and accelerates model convergence. Experiments 5–8 demonstrate that combining multiple modules yields further performance gains across all metrics. The synergistic effects of RCS_M and ODConv in feature enhancement, combined with the multi-scale fusion capability of BiFPN, prove particularly effective in dense, heavily occluded scenarios by improving localization accuracy for previously difficult vehicle instances.

#### 4.3.4. Classification Accuracy Evaluation

**Figure 10:**
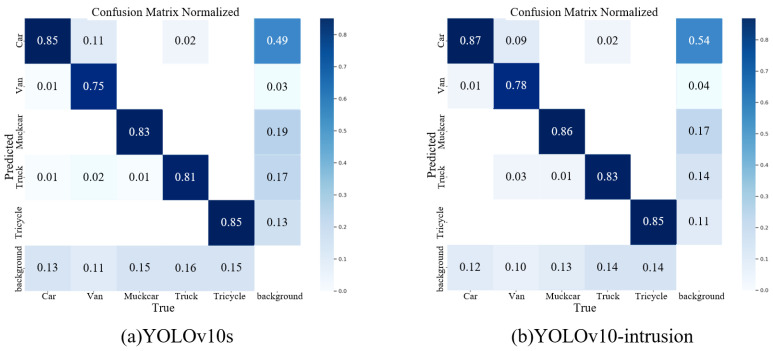
Confusion Matrices of Different Models on the Self-Built Dataset YOLOv10s.

The confusion matrices of YOLOv10s and YOLOv10-intrusion generated on the vehicle area intrusion detection dataset are shown in Figure 10.

As can be seen from Figure 10a, for YOLOv10s, the classification accuracies of car, van, muck car, truck, and tricycle are 85%, 75%, 83%, 81%, and 85%, respectively. As shown in Figure 10b, for YOLOv10-intrusion, the classification accuracies of car, van, muck car, truck, and tricycle reach 87%, 78%, 86%, 83%, and 85%, respectively. Compared with YOLOv10s, the accuracies of the first four categories are improved by 2%, 3%, 3%, and 2%, respectively. In addition, van has a 9% probability of being misclassified as car, which is 2% lower than that of YOLOv10s. Meanwhile, car no longer has a 1% probability of being misclassified as truck, representing a 1% reduction compared with YOLOv10s. In summary, the YOLOv10-intrusion model can effectively improve classification accuracy and alleviate the problems of misclassification and missed detection.

#### 4.3.5. Visualization Analysis

**Figure 11:**
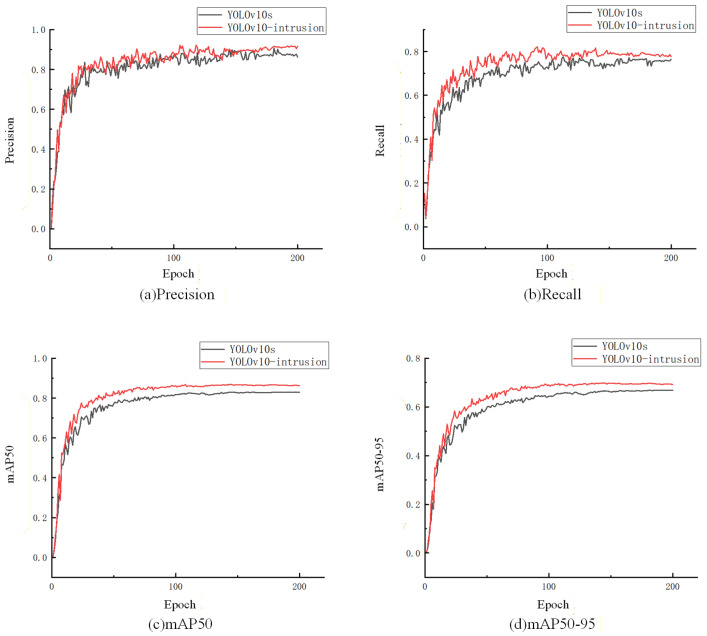
Performance metric comparison between YOLOv10-Intrusion and baseline YOLOv10s.

As illustrated in Figure 11, YOLOv10-Intrusion consistently outperforms the baseline YOLOv10s across all evaluation metrics: precision improves by 1.5 percentage points, recall by 3.3 percentage points, mAP@0.5 by 3.6 percentage points, and mAP@0.5:0.95 by 2.8 percentage points.

**Figure 12:**
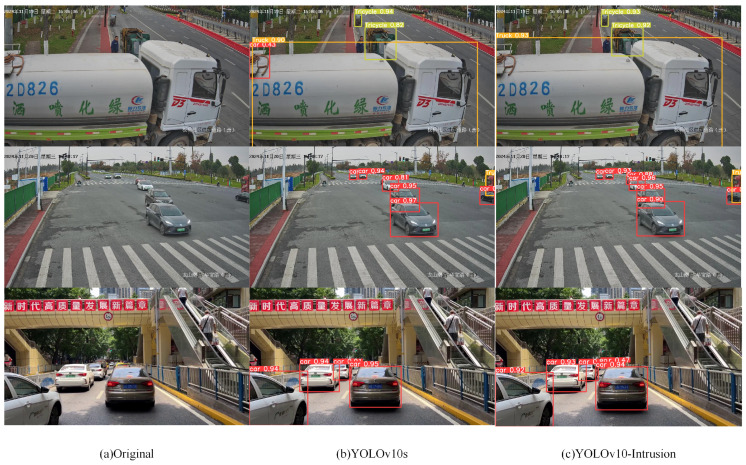
Detection result comparison before and after model improvement. (The text in the picture represents the time and location of the shooting).

To provide a more direct visual illustration of the detection improvements, representative road section scenes comparing baseline and improved model outputs are shown in Figure 12. Columns from left to right correspond to the original image, the YOLOv10s detection result, and the YOLOv10-Intrusion detection result. Row 1 presents a close-range large vehicle detection scenario; Rows 2 and 3 present scenes with dense target occlusion.

In Scene 1, YOLOv10s misidentified the rear of a large vehicle as a passenger car. In Scenes 2 and 3, YOLOv10s failed to detect occluded vehicles, causing missed detections. The improved model reduces inter-class misclassification of medium and large vehicle categories while achieving higher detection confidence for occluded instances, thereby enhancing detection stability and reducing missed detections in the vehicle area intrusion detection task.

#### 4.3.6. Field Validation

**Figure 13:**
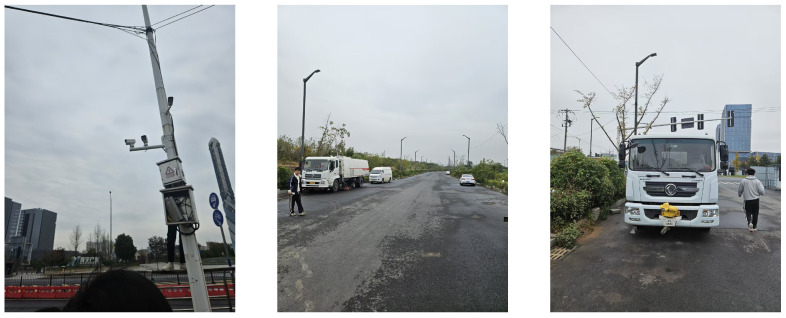
Field tests on monitored road sections.

Following preliminary online evaluation, field tests were conducted to validate detection performance under real-world conditions. The test procedure involved physically driving a test vehicle along the monitored road sections to simulate illegal intrusion events. Each test session covered three stages: simulating camera orientation perturbations to assess robustness to environmental disturbances; traversing the legal driving zone to verify the absence of false alerts; and entering the designated intrusion zone to confirm real-time detection and alert triggering. Field test scenarios are shown in Figure 13.

The first two test sections were located on high-traffic main roads with cameras primarily capturing vehicle side profiles. When the test vehicle entered the pedestrian path from a distance, no alert was initially triggered because only the front section had entered the detection zone—an issue resolved after dataset refinement. The third and fourth sections were low-traffic forest roads with frontal camera perspectives, where immediate detection was achieved even for small vehicle front intrusions. The fifth and sixth sections were construction site entrances with rear-focused cameras, where alerts were rapidly triggered after vehicle entry. All six sections achieved satisfactory detection performance in daytime evaluation. Nighttime tests conducted with large vehicles successfully replicated all daytime scenarios, achieving complete detection coverage across all simulated intrusion events.

**Figure 14:**
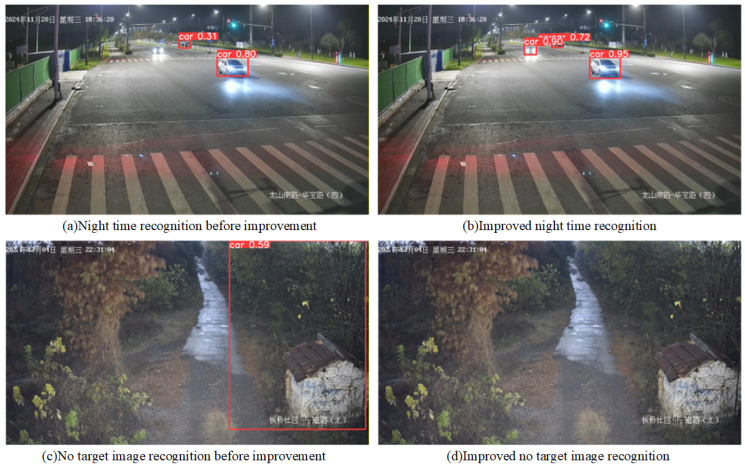
Comparison of nighttime and no-target misdetection before and after dataset improvement. (The text in the picture represents the time and location of the shooting).

Three rounds of iterative dataset refinement were performed. In the first round, additional side-profile images and targeted augmentation were incorporated to address underrepresentation of small vehicle side views, resolving the false negatives at the first two sections. In the second round, ODConv was introduced and low-quality large vehicle images were filtered to address nighttime truck/muck-truck misclassification; recall consequently improved by 1.3 percentage points, and the detection threshold was raised from 0.5 to 0.6 without significant missed detections. In the third round, representative background and transmission artifact images were added to suppress false alerts, improving precision by 0.6 percentage points. Figure 14 compares detection results before and after dataset improvement.

Before improvement, detected vehicle confidence scores were low and one headlight-occluded vehicle was missed. After refinement, more vehicles were detected with higher confidence scores, demonstrating the effectiveness of the iterative dataset improvement in reducing missed detections under challenging nighttime conditions.

## 5. Conclusions

This paper proposes YOLOv10-Intrusion, an improved object detection algorithm based on YOLOv10s for vehicle area intrusion detection in real-world road surveillance environments. To address prevalent false detection and missed detection challenges, a dedicated vehicle area intrusion dataset was constructed from field surveillance footage, covering five vehicle categories across diverse road environments and lighting conditions.

The proposed algorithm integrates four complementary architectural improvements. The self-designed RCS_M module replaces the backbone C2f structure to enhance cross-channel information exchange, local feature representation, and cross-scale interaction capability. ODConv (C2f_OD) replaces standard convolution in the neck C2f module to strengthen fine-grained recognition of visually similar vehicle categories. BiFPN replaces the original neck feature fusion structure to adaptively balance multi-scale feature contributions and improve recall in dense, occluded scenarios. WIoU replaces the CIoU loss function to improve bounding box regression stability and convergence speed while alleviating the adverse effects of class imbalance.

Experimental results and field testing demonstrate the capacity of YOLOv10-Intrusion to accurately identify illegal vehicles in real-world surveillance environments, meeting practical enforcement requirements. Compared with the baseline YOLOv10s, the proposed algorithm achieves improvements of 1.5, 3.3, 3.6, and 2.8 percentage points in precision, recall, mAP@0.5, and mAP@0.5:0.95, respectively, and outperforms other mainstream object detection algorithms in terms of both model accuracy evaluation metrics and actual deployment effectiveness.

Future work will explore model compression techniques (pruning and knowledge distillation) to reduce parameter count and improve deployment efficiency under limited computational resources. Additionally, extending the platform to support multi-camera joint detection and incorporating nighttime-enhanced imaging modalities will further improve intrusion detection robustness under adverse environmental conditions.

## Figures and Tables

**Table 1:** Category distribution of the vehicle area intrusion dataset.

Category	Instance Count
Car	7413
Van	603
Muck Car	1594
Truck	1886
Tricycle	964
Total	12,460

**Table 2:** Experimental software and hardware configuration.

Configuration	Specification
Operating System	Windows 11
Programming Language	Python 3.9
Deep Learning Framework	PyTorch 2.0.1
GPU Acceleration	CUDA 11.3
CPU	Intel Xeon Platinum 8350C
System RAM	56 GB
GPU	NVIDIA RTX 3090 (24 GB VRAM)

**Table 3:** Experimental parameters.

Parameter	Parameter Value
Epoch	200
BatchSize	8
Ir0	0.01
Momentum	0.937
Weight decay	0.0005

**Table 4:** Performance comparison of different algorithms on the vehicle area intrusion dataset.

Model	P (%)	R (%)	mAP50 (%)	mAP50:95 (%)	Params (M)	GFLOPs	FPS
YOLOv10-Intrusion (Ours)	89.2	78.6	86.6	69.7	11.5	29.9	123
Faster R-CNN	78.8	65.2	71.2	59.1	137.0	370.2	32
SSD	79.1	63.1	73.3	59.3	24.8	275.4	57
YOLOv8s	84.3	71.9	82.1	65.8	11.1	28.6	127
YOLOv11s	87.1	75.1	82.8	67.3	9.4	21.5	144
YOLOv12s	86.3	74.7	82.4	67.1	9.1	19.7	148
RTDETR-L	84.5	76.6	81.5	63.0	31.0	108.3	76
Deformable DETR	80.6	72.1	77.8	61.2	39.8	97.6	83
YOLO-LCR	82.1	70.2	81.1	64.4	12.4	40.4	90
TSA-YOLO	84.8	71.9	82.6	67.7	17.4	55.6	83

**Table 5:** Generalization experiment.

Dataset	Model	mAP50 (%)	mAP50:95 (%)	Params (M)	GFLOPs	FPS
KITTI	YOLOv10s	83.8	60.4	8.1	24.6	122
KITTI	YOLOv10-intrusion	86.0	62.2	11.5	29.9	116
VOC2007	YOLOv10s	82.3	59.2	8.1	24.6	107
VOC2007	YOLOv10-intrusion	84.1	61.8	11.5	29.9	102
COCO	YOLOv10s	55.6	36.4	8.1	24.6	97
COCO	YOLOv10-intrusion	61.0	40.2	11.5	29.9	93

**Table 6:** Ablation experiment results on the vehicle area intrusion dataset. (× indicates that the module has not been used, ✓ indicates the use of the module).

Exp.	C2f_OD	RCS_M	BiFPN	WIoU	P (%)	R (%)	mAP50 (%)	mAP50:95 (%)	Params (M)	GFLOPs	FPS
0	×	×	×	×	87.7	75.3	83.0	66.9	8.1	24.6	142
1	✓				86.7	76.6	83.0	67.0	8.6	25.8	134
2		✓			87.6	75.5	85.3	68.6	10.6	27.9	128
3			✓		88.4	76.9	83.9	67.5	8.5	25.4	137
4				✓	87.3	77.7	83.6	67.4	8.1	24.6	142
5	✓	✓	✓		87.9	76.6	85.0	67.8	11.5	29.9	123
6		✓	✓	✓	89.7	76.2	85.6	68.9	11.0	28.7	130
7	✓	✓		✓	88.6	77.3	85.4	68.5	11.1	29.1	126
8	✓	✓	✓	✓	89.2	78.6	86.6	69.7	11.5	29.9	123

## Data Availability

The data presented in this study are available at https://github.com/MrFENGXINGYU/vehicle-intrusion-det (accessed on 4 March 2026).
